# Does Oxygen Work? Evidence for Oxygenation During Kidney Graft Preservation: A Review

**DOI:** 10.3390/jcm14061927

**Published:** 2025-03-13

**Authors:** Andres Calva Lopez, Jose Enrique Robles Garcia, Carlos Andres Yanez Ruiz, Mario Daniel Tapia Tapia, Vanessa Talavera Cobo, Carmina Alejandra Muñoz Bastidas, Bernardino Miñana Lopez, Daniel Sanchez Zalabardo

**Affiliations:** Department of Urology, Clínica Universidad de Navarra, 31008 Pamplona, Spain; acalva@unav.es (A.C.L.); cyanezruiz@unav.es (C.A.Y.R.); mdtapia@unav.es (M.D.T.T.); vtalavera@unav.es (V.T.C.); cmbastidas@unav.es (C.A.M.B.); bminana@unav.es (B.M.L.); dsanchezz@unav.es (D.S.Z.)

**Keywords:** kidney transplantation, kidney graft preservation, oxygenation, ischemia-reperfusion injury, oxygen carriers, hyperbaric oxygenation, two-layer method, retrograde oxygen persufflation, hypothermic oxygenated machine perfusion, delayed graft function

## Abstract

Kidney transplantation (KT) is the gold-standard treatment of end-stage kidney disease (ESKD). Traditional preservation methods, such as static cold storage (SCS), have been replaced by modern and more effective preservation methods, especially hypothermic machine perfusion (HMP). Regardless of improved preservation, ischemia-reperfusion injury (IRI) is inevitable, limiting graft functionality through delayed graft function (DGF) and graft survival. Supplementing the ischemic kidney graft with oxygen during hypothermic preservation has been used in different methods as an attempt to counteract IRI and its effects on graft function and survival. Various oxygenation methods have been studied, from adaptations of classic and well-known preservation strategies, like the addition of oxygen carriers to SCS, or more innovative preservation methods, like hyperbaric oxygenation or retrograde oxygen persufflation. In this review, we will attempt to provide a summary of the available evidence on oxygen carriers, hyperbaric oxygenation, the two-layer method, retrograde oxygen persufflation, and hypothermic oxygenated machine perfusion (HOPE) and discuss the effect these strategies have on kidney graft functionality.

## 1. Introduction

Kidney transplantation (KT) is the established gold-standard treatment for end-stage kidney disease (ESKD). KT improves ESKD patient survival, improves their quality of life, lowers the burden on healthcare systems worldwide since it is a much more cost-effective measure, and reduces mortality associated with other forms of renal replacement therapy (RRT), such as hemodialysis. Due to population aging, ESKD is expected to increase, accounting for a concerningly increasing percentage of mortality worldwide [1]. However, the prime limitation to lessen the morbimortality associated with ESKD and RRT is the limited number of deceased kidney donors (DKDs) and the varying quality of the procured kidney grafts. Over the past decades, new groups of DKDs have been established as a means to counteract the lack of DKDs, with the use of donors after circulatory death (DCDs) and expanded-criteria donors (ECDs), leading to increased kidney graft availability for ESKD patients. Nonetheless, with a continuously aging population, DKDs become suboptimal donors and kidney graft viability comes into question. Kidney graft preservation has primarily relied on the concept of hypothermia, reducing kidney graft metabolism and preventing graft tissue degradation by enzymatic deregulation, by almost 3-fold for every 10 °C reduction in kidney graft core temperature. With hypothermic preservation, graft metabolism still remains at 10%, making an argument for oxygen supplementation during preservation to ensure adequate mitochondrial synthesis of adenosine triphosphate (ATP), upholding aerobic cellular respiration in an attempt to delay the kidney graft injury process [2]. Even so, cold ischemia time (CIT) still disrupts viable kidney graft tissue, interfering with short-term graft function by playing a key role in the development of delayed graft function (DGF) and long-term graft function, reducing overall graft survival.

In this review, we present different methods of oxygen supplementation during kidney graft preservation, including oxygen carriers, hyperbaric oxygenation, the two-layer method, retrograde persufflation, and hypothermic oxygenated machine perfusion. We will outline these methods and their effects on short- and long-term kidney graft outcomes, as well as discuss the latest evidence for each preservation method.

## 2. Ischemia-Reperfusion Injury

Ischemia-reperfusion injury (IRI) is an unavoidable process which occurs after the blood supply is temporality halted and then reestablished to a functioning organ. Conditional to the graft procurement setting, kidney grafts can suffer cold or warm ischemia. Cold ischemia occurs after graft procurement and preservation, while warm ischemia occurs during vascular anastomosis during kidney transplantation. A reduced blood supply triggers a series of biological pathways, disrupting cellular metabolism, due to the graft’s inability to remove waste products in the absence of adequate oxygen levels. These changes induce the following [3,4]:

(a) Decreased ATP synthesis;
(b) Increased lactic acid production (resulting in intracellular acidosis);
(c) Decreased Na^+^/K^+^ ATPase, Na^+^/H^+^ ATPase, and Ca^2+^ ATPase pump activity, causing Na^+^/K^+^ and Na^+^/H^+^ imbalance (resulting in intracellular edema and acidosis) and an increased intracellular Ca^2+^ concentration;
(d) Cell cycle dysregulation;
(e) Cytoskeletal protein disruption.

Increased Na^+^ and Ca^2+^ intracellular concentrations activate Ca^2+^-dependent proteases (calpains) and lysosomal enzymes, which paradoxically become inactive under an acidic environment. Upon reperfusion, the incoming blood flow modifies ischemic conditions, adjusting the intracellular pH and allowing calpains and lysosomal enzymes to become fully active and produce tissular damage [5]. The reinstatement of blood flow and the corresponding increased oxygen levels under ischemic conditions are accountable for the production of harmful reactive oxygen species (ROS), particularly superoxide anions. Ca^2+^ and ROS surplus, together with mitochondrial dysfunction, increase mitochondrial permeability and release cytotoxic molecules, which induce tissular necrosis, apoptosis, and necroptosis [5,6].

## 3. Methods of Oxygenation

Ensuring an adequate oxygen concentration during graft preservation mitigates the effect of IRI on graft function. Due to the various metabolic changes induced by oxygen depletion, maintaining oxygen levels within graft tissue could counteract intracellular edema and acidosis as a result of ion disbalance. Additionally, it prevents ROS formation after the oxygenated blood supply is restored during KT.

### 3.1. Oxygen Carriers

**Figure 1:**
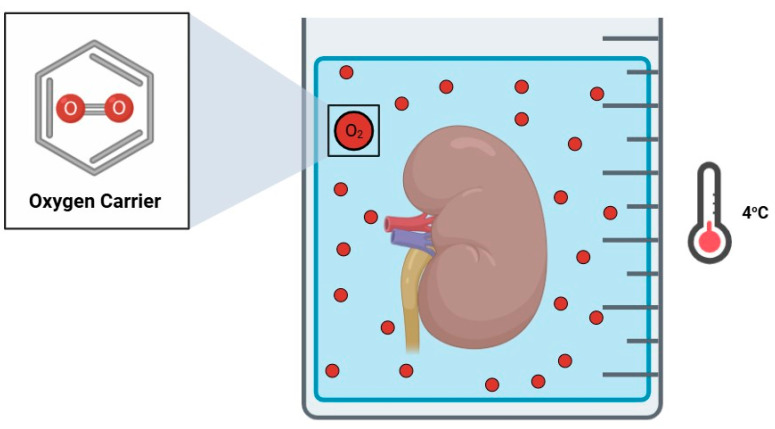
Static cold storage supplemented with an oxygen carrier.

Oxygen carriers (OCs) are molecules capable of transporting oxygen in a medium. Due to their simple implementation using a well-established preservation protocol, by complementing a preservation solution with OCs, they act as an attractive addition, especially for static cold storage (SCS) kidney preservation, to supplement kidney grafts with oxygen in an attempt to improve graft quality (Figure 1). M101 is extracellular hexagonal bilayer hemoglobin, a type of respiratory pigment extracted from *Arenicola marina*, a lugworm living under the sand on beaches, and continuously exposed to normothermia/normoxia and hypothermia/hypoxia conditions, depending on the sea tides, mimicking the process of graft procurement, preservation, and implantation. M101 possesses the following advantages which allow it to adequately perform as an OC [7,8]:

(a) A high oxygen affinity and capacity (up to 156 O_2_ molecules);
(b) Gradient-based oxygen release (a stable oxygen-rich environment);
(c) A wide temperature range (4 °C to 37 °C);
(d) A lack of immunogenicity.

Thuillier et al. developed an in vitro model of porcine proximal tubule cell cultures and a porcine kidney autotransplantation model with grafts preserved by SCS, studying the effect of 1 g/L and 5 g/L M101 supplementation, respectively. They observed improved preservation with M101-supplemented cell culture lines, with lower concentrations of cellular injury biomarkers, including a reduction in lactate dehydrogenase (LDH) release. In the autotransplantation model, M101-supplemented porcine kidney grafts maintained urine production and had lower creatinine levels, faster graft function recovery, and a reduced inflammatory/immune response [9]. The same study group developed a similar porcine kidney autotransplantation model, with M101 supplementation at increasing concentrations (1, 2, and 5 g/L), with increased graft function recovery and lower creatinine levels when compared to SCS 1 week and 3 months after autotransplantation [10].

Attributable to its preclinical successful results, M101 has become commercially available as HEMO_2_Life^®^ (Hemarina, Morlaix, France), with more studies attempting to determine the benefits of OC supplementation for kidney graft preservation. As a consequence of an increasing marginal donor population, which requires optimal kidney graft preservation, different groups have studied the effect of OCs when added to a hypothermic machine perfusion (HMP) circuit. Kaminski et al. developed a DCD porcine autotransplantation model, evaluating the effect of additional HEMO_2_Life^®^ added to HMP compared to that of HEMO_2_Life^®^ added to SCS. Their results suggest the addition of HEMO_2_Life^®^ to HMP improves the short-term and long-term results of kidney grafts, with lower creatinine levels and improved graft function 1 month and 3 months after transplantation [11]. Similarly, Kamil et al. developed a DBD porcine autotransplantation model with the supplementation of 100% O_2_ at 1 L/min, as well as the addition of HEMO_2_Life^®^, with HMP as the selected preservation method. Their results suggest HEMO_2_Life^®^ supplementation, regardless of the addition of 100% O_2_, improves kidney graft function 3 months after transplantation [12].

Promising preclinical and animal studies’ results have allowed for the use of HEMO_2_Life^®^ supplementation in human kidney transplantation. In 2020, Le Meur et al. published the first-in-human study of HEMO_2_Life^®^, the OXYgen carrier for Organ Preservation (OXYOP) study. It consisted of 60 DKDs, preserved by SCS in case of standard criteria donors (SCDs) or HMP in case of ECDs, with grafts paired to be treated with HEMO_2_Life^®^ or left untreated prior to implantation. The primary outcome for the OXYOP study was safety, finding no differences in adverse effects among both groups at the 1-year follow-up. Secondary outcomes included patient survival, graft survival, graft function, and graft loss; the results found comparable patient and graft survival rates and non-significantly reduced rates of DGF in the HEMO_2_Life^®^-treated grafts. These results indicate that HEMO_2_Life^®^ is a safe tool for kidney graft preservation [13], with later studies continuing to provide evidence that HEMO_2_Life^®^ reduces delayed graft function rates, independent of cold ischemia times [14]. Le Meur et al. attempted to provide high-quality evidence regarding the effect of HEMO_2_Life^®^ with the development of OXYgen carrier for Organ Preservation 2 (OxyOp2), a prospective, multicenter, randomized clinical trial (RCT) (NCT04181710) that recruited a total of 450 patients and concluded in September 2023; the results are yet to be published [15].

### 3.2. Hyperbaric Oxygenation

**Figure 2:**
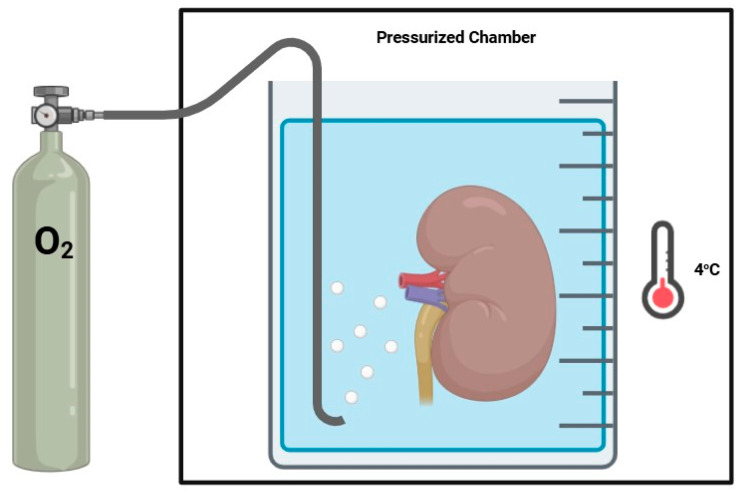
Hyperbaric oxygenation.

Hyperbaric oxygenation (HO) consists of the delivery of oxygen while under supraphysiological atmospheric pressures (Figure 2). Nowadays, HO is used in the treatment of carbon monoxide poisoning, gaseous embolisms, and decompression sickness, as well as to promote wound healing. At the average atmospheric pressure or one atmosphere (1 atm), blood oxygenation is limited. Increasing atmospheric pressure allows for an increased level of dissolved oxygen in the blood, permitting higher tissular oxygenation, even in the absence of adequate vascularization, allowing HO to be seen as potentially advantageous in organ preservation.

HO in kidney graft preservation has been studied since the 1960s, with a series of successful canine autotransplantation models, with the preservation of grafts at varying pressures (3–15 atm) and under hypothermia [16,17,18,19,20]. There is little evidence regarding the use of HO as a preservation method in human kidney transplantation. In 1965, Manax et al. published two cases of successful human kidney transplants after preservation by HO (3 and 5 atm) and hypothermia (2 °C); although both recipients died 2 weeks after the transplantation because of complications, the autopsy results proved that both kidney grafts were viable, with no macroscopic or microscopic signs of graft rejection [17]. Even though HO has not been widely used as a preservation method, recent evidence shows that HO is beneficial in mitigating autophagy dysfunction and inflammatory responses in IRI after transplantation [21] or as an organ preservation method in DBDs before organ procurement [22]

### 3.3. Two-Layer Method

**Figure 3:**
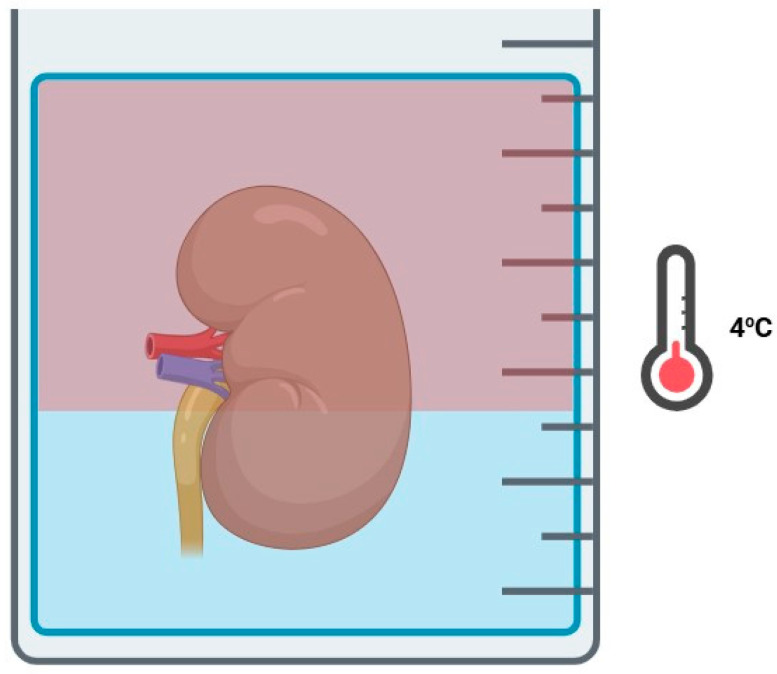
Two-layer method.

The two-layer method (TLM) in kidney graft preservation involves submerging the graft in a mixture of two solutions with different densities, allowing the graft to remain in contact with both layers (Figure 3). Perfluorocarbons (PFCs), a key component of TLM, are a type of inert solutions with high capacity for dissolved oxygen, which work similarly as OC by releasing O_2_ molecules against a gradient and remain unaffected by temperature changes. PFCs’ density differences compared to standard preservation solutions make them particularly useful for TLM applications.

Evidence shows that the most successful application of PFCs in graft preservation, especially through the TLM, is with pancreas preservation by permitting a sufficient amount of ATP synthesis to ensure graft viability. However, little is known on the effect of PFCs and the TLM as a preservation method for kidney grafts [23]. An initial preclinical in vitro study by Maluf et al. compared molecular biomarkers in rodent kidney grafts after a 24 h preservation period with SCS or the TLM, finding elevated levels of heme oxidase-1 (HO-1), with known cytoprotective effects, and reduced inducible nitric oxide synthases (iNOS). These changes increase reactive nitric oxide species synthesis, which have detrimental effects on kidney graft function [24]. Marada et al. published similar findings in a rat transplantation model, with increased 1-month survival rates, lower 24 h creatinine levels, and less tissular damage when comparing the TLM with SCS [25]. In contrast, in a porcine kidney graft preservation model using isolated organ preservation systems, Hosgood et al. reported lower renal blood flow, increased renal resistance, lower creatinine clearance, and increased levels of oxidative and inflammatory biomarkers in grafts preserved by the TLM compared to SCS [26]. Owing to very limited and conflicting preclinical evidence, human kidney graft preservation by the TLM remains uncertain and with limited applications.

### 3.4. Retrograde Oxygen Persufflation

**Figure 4:**
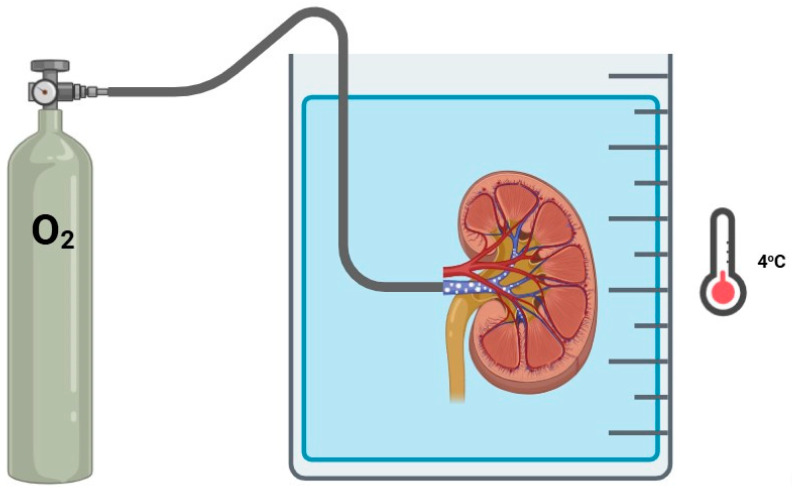
Retrograde oxygen persufflation.

Retrograde oxygen persufflation (ROP), also known as venous systemic oxygen persufflation, is an innovative technique which delivers pure, filtered and humidified oxygen to a kidney graft directly through the renal vein. This method involves creating small perforations on the renal cortex to allow for oxygen release while maintaining low intrarenal pressures (Figure 4). Early in-human experience with persufflation occurred by a malfunctioning oxygenated perfusion circuit. Flatmark et al. published a series of 4 human kidney grafts that underwent unintentional persufflation, finding preserved primary function of all grafts, despite significantly increased intrarenal resistance and decreased renal flow [27].

ROP was studied in the 1980s with a series of animal autotransplantation models. In a canine autotransplantation model, Rolles et al. found graft viability and function after up to 48 h of ROP preservation in grafts with up to 60 min of warm ischemia [28]. The same group developed a similar rabbit autotransplantation model with evidence of increased ATP production and consumption in ROP-preserved grafts, along with mitochondria structural preservation, when compared to SCS [29]. Treckmann et al. made an attempt to further explore ROP with porcine autotransplantation models and demonstrated preserved graft function after 60, 90, and 120 min of warm ischemia when preserved by ROP 4 h prior to autotransplantation; however, the graft survival was comparable to that of SCS [30]. A follow-up study compared ROP with HMP and SCS showing 100%, 60%, and 57% graft survival, respectively, 1 week after transplantation [31]. These results, although limited, demonstrate ROP as a promising preservation method, especially for DCD kidney grafts, with other animal studies finding similar results [32,33,34].

These hopeful results allowed for a human pilot study of 10 DKDs with paired grafts preserved by ROP or SCS. They found apparent lower creatinine levels 2 weeks after transplantation and lower rates of graft loss after significant warm ischemia times, but no statistical analysis was performed to compare these results [35]. Due to advancements in alternative preservation methods, ROP remains unexplored in human kidney graft preservation. High-quality evidence is warranted to establish its efficacy and potential advantages over other preservation methods.

### 3.5. Hypothermic Oxygenated Machine Perfusion

**Figure 5:**
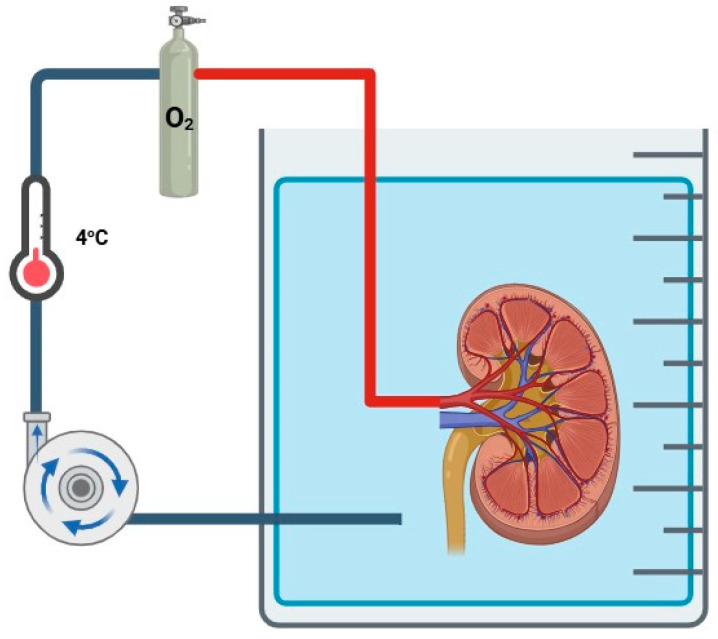
Hypothermic oxygenated machine perfusion.

HMP consists of a continuous perfusion of a cold preservation solution at a low temperature (2 °C to 8 °C). It provides a continuous flush of renal vasculature, removing waste metabolites, and ensures adequate microcirculation within the graft. It has been characterized as superior to SCS in preventing DGF, irrespective of donor types [36,37,38,39,40,41,42]. Hypothermic oxygenated machine perfusion (HOPE) consists of oxygen supplementation during HMP (Figure 5), theoretically increasing the well-known benefits of HMP by replenishing ATP levels during ischemia.

#### 3.5.1. Animal Studies

Prior to the implementation of HOPE in human kidney transplant programs, a series of animal studies and autotransplantation models have emerged as a means of proving the theoretical effect of oxygen supplementation, in addition to the validated benefits of HMP. As early as 2004, Minor et al. studied the effect of HOPE in DCD porcine kidneys by comparing HOPE with two different solutions: Belzer machine perfusion solution (MPS) and histidine–tryptophan–ketoglutarate (HTK) MPS, also known as Custodiol^®^ (Essential Pharmaceuticals, Ewing Township, NJ, USA), with SCS. A molecular analysis determined higher levels of ATP synthesis in HOPE-preserved grafts than those preserved by SCS, with even better results using Belzer MPS (Custodiol^®^–HOPE ATP 1.18 mmol/g; Belzer–HOPE ATP 2.43 mmol/g; SCS ATP 0.16 mmol/g; *p* < 0.05) [43]. This was one of the first studies to demonstrate the positive impact of ATP synthesis in kidney grafts due to oxygen supplementation. However, it did not compare graft function between groups. In 2015, the same group developed a porcine autotransplantation model which assessed the effect of different MPSs during HOPE, comparing Custodiol^®^ with KPS-1^®^ (Organ Recovery Systems, Itasca, IL, USA) and finding statistically significant differences favoring Custodiol^®^ over KPS-1^®^, as they reported less tubular damage in graft biopsies and increased graft function in the first 24 h after successful transplantation (*p* < 0.05) [44]. This study further demonstrated an apparent superiority of Custodiol^®^ during HOPE over other types of perfusion solutions.

Doorschodt et al. also developed a porcine autotransplantation model attempting to compare SCS to HOPE, with the use of the novel Airdrive^™^ perfusion system (Indes, Enschede, Netherlands). Similar to previous animal studies, they found statistically significant lower creatinine levels after autotransplantation in HOPE-preserved grafts (*p* < 0.001), as well as less biopsy-proven tubular damage and tissular edema, with SCS-preserved grafts showing glomerular shrinkage and inflammatory infiltration in biopsies taken after transplantation [45]. Unlike similar studies, this group assessed kidney graft microcirculation with a combined laser Doppler and flowmetry device, at 2 mm and 8 mm tissular depths, finding increased capillary blood flow and velocity in HOPE-preserved kidney grafts (*p* < 0.05), as well as a more homogenous perfusion directly after revascularization in HOPE-preserved grafts when compared to SCS [45]. The presence of a homogeneous perfusion after revascularization indicates that the benefits of HMP are still present and are not mitigated by oxygen supplementation, allowing for a generalized microvasculature dilation [46], a phenomenon not observed with SCS.

In an attempt to simulate human kidney transplantation scenarios, Buchs et al. studied the effect of HOPE in DCD porcine kidney grafts, with and without the presence of 30 min of warm ischemia, compared to traditional SCS preservation. Kidney grafts without warm ischemia and immediately preserved by HOPE showed replenished ATP levels after 8 h and 18 h preservation periods, with little variation between both measurements; however, when undergoing 30 min of warm ischemia, the HOPE-preserved grafts presented lower ATP levels when compared to the ischemia-free grafts. In the case of SCS, grafts with and without ischemia did not recover ATP levels [47]. These results propose HOPE as a preservation technique able to counteract the effects of warm ischemia. Similarly, in 2016, Kron et al. assessed the effect of 30 min of warm ischemia with the use of HOPE or SCS in DCD rat kidney grafts after autotransplantation. In this model, HOPE was instated for 1 h after SCS, prior to autotransplantation, finding increased ATP levels, lower creatinine levels, and a lower ROS concentration when compared to SCS [48]. A small group of rat kidney grafts in this study underwent normothermic oxygenated perfusion in an attempt to compare hypothermic oxygenation with normothermic oxygenation. Interestingly enough, functional results with regards to ATP and creatinine levels favored HOPE over normothermic oxygenation [48].

Darius et al. designed a porcine autotransplantation model in an attempt to assess the effect of various dynamic preservation strategies, with different combinations of SCS, HMP, or HOPE. Porcine kidney grafts underwent 30 min of warm ischemia and were randomized to different 22 h preservation strategies. Regardless of the addition of oxygen, the continuous HMP-preserved grafts had increased early graft function when compared to SCS and all of the end-ischemic strategies. Continuous HOPE displayed significant differences in creatinine levels when compared to continuous HMP, as well as increased renal flow and lower renal resistance during preservation [49]. The same group assessed the effect of different oxygen partial pressures (pO_2_) (low pO_2_ at 30% and high pO_2_ at 90%) during continuous HOPE, finding significant differences in perfusion solution pO_2_ (a three-fold increase in low pO_2_ levels and 10-fold increase in high pO_2_ levels) and metabolic measures, which did not translate to differences in functional results [50]. Darius et al. also explored novel oxygenation techniques, assessing the impact of bubble and intermittent surface oxygenation, rather than membrane oxygenation, which is more widespread. Using the same autotransplantation model, they assessed the effect of direct bubble and 30 min surface oxygenation at the start of HMP and 1 h end-ischemic oxygenation before implantation. This innovative oxygenation strategy allowed for maintained supraphysiological pO_2_ levels within the preservation solution as well as an increase in ATP levels [51]. These findings have demonstrated that multiple and combined preservation strategies are a viable option, in addition to proposing an emerging alternative oxygenation technique, eliminating the need for external membrane oxygenation and reducing the overall costs of HOPE in KT programs.

Animal studies regarding the use of HOPE in different modalities of graft transplantation ensure the safety of oxygen supplementation during HMP, with apparent better functional results, as well as show the theoretical proposed benefits of HOPE, mainly increased ATP synthesis, regardless of the presence of warm ischemia. This allows us to consider HOPE as a feasible and practical preservation method, independent of the type of donor (DBD, DCD, etc.), which could benefit KT recipients in human KT programs.

#### 3.5.2. Human Clinical Studies

Promising results in animal studies have allowed for HOPE to transition from animal autotransplantation models to human KT programs. Some of the initial studies implementing HOPE in humans recommended short periods of oxygen supplementation during standard HMP, rather than continuous oxygenation, proclaiming oxygen supplementation in human kidney grafts as safe for grafts and recipients, as well as improving overall graft function and reducing inflammatory cytokines and immune responses [52,53]. As of today, there is limited high-quality evidence regarding the use of HOPE in human KT programs (Table 1), mainly because of the overwhelming evidence supporting HMP and its effect on graft function [36,37,38,39,40,41,42].

The COMPARE trial [54], a landmark European multicenter, randomized, and double-blind clinical trial, was one of the first and largest studies comparing HOPE to HMP in DCD kidney grafts from donors who were 50 years old or older, with a total of 106 paired kidneys included in the study. Oxygen supplementation was achieved in the intervention group with supraphysiological 100% oxygen delivery via a membrane oxygenator at a flow of 100 mL/min O_2_, using the Kidney Assist™ Transporter (Organ Assist, Gothenburg, Sweden). The primary outcome was defined as the 1-year post-transplantation estimated glomerular filtration rate (eGFR), while the secondary outcomes were recipient and graft survival at 1-year post-transplantation, primary non-function (PNF) (defined as the reinstitution of sustained RRT), DGF, functional DGF (defined as the absence of a decrease in creatinine levels by at least 10% per day over 3 consecutive days), eGFR 3 months and 6 months after transplantation, creatinine clearance at 1-year post-transplantation, acute graft rejection confirmed by biopsy up to 1 year after transplantation, and safety events. Jochmans et al. [54] found statistically significant differences for the primary outcome at 12 months after transplantation (47.6 mL/min/1.73 m^2^ in the HOPE group vs. 42.6 mL/min/1.73 m^2^ in the HMP group; *p* = 0.035). However, when only considering paired grafts that were both functioning after follow-up (*n* = 83), there were no differences in the eGFR 1 year after KT (50.5 mL/min/1.73 m^2^ in the HOPE group vs. 46.7 mL/min/1.73 m^2^ in the HMP group; *p* = 0.12). Moreover, they found statistically significant differences in secondary outcomes, including less severe complications (Clavien–Dindo grade III or more) (11% vs. 16%; *p* = 0.032), lower rates of kidney graft failure at 12 months (3% vs. 10%; *p* = 0.028), and a 44% reduction in biopsy-proven acute kidney graft rejection at 3 months (14% vs. 26%; *p* = 0.040), all in favor of HOPE. Regardless of the primary outcome results, the COMPARE trial establishes the cost-effectiveness of oxygen supplementation to HMP, associating the low additional cost of HOPE and considerable benefits, including less severe complications, shorter hospital stays, fewer rates of acute rejection, and overall improved graft survival; thus, improving recipient outcomes and reducing healthcare costs to KT programs.

Recent studies have provided valuable insights into the effect of oxygen supplementation during HMP in KT. Houtzager et al. developed a small clinical pilot study of five DKDs (four DBDs and one DCD) using the Airdrive^™^ perfusion system, with the primary outcomes being adverse events and safety, and found no significant differences 1 month after transplantation [55]. Meister et al. compared a total of 15 ECD kidney grafts undergoing end-ischemic HOPE (end-HOPE) using the Kidney Assist™ Transporter with 30 matched kidney grafts preserved by SCS. They also found no significant differences regarding the primary outcome (DGF; 53% vs. 33%; *p* = 0.197) 6 months after transplantation; however, the relative decrease in renal vascular resistance during end-HOPE showed a significant inverse association with creatinine levels (r = −0.682; *p* = 0.006). This inverse association was more prominent in grafts that developed PNF compared to grafts that developed DGF (*p* = 0.013) [56]. Ravaioli et al. published a small Italian clinical trial comparing end-HOPE to SCS in a total of 10 ECD kidney grafts, matched with control ECD kidney grafts at a ratio of 1:3. They found no significant differences regarding safety and adverse events (primary outcome) or PNF and graft survival (secondary outcomes). The incidence of DGF was different in both groups (20% in end-HOPE vs. 40% in SCS), but when only considering end-HOPE-preserved kidney grafts, those that underwent end-HOPE for more than 2 h showed no DGF compared to grafts with shorter perfusion times (0% vs. 40%; *p* = 0.04) [57].

Husen et al. published a large European, multicenter, randomized clinical trial, the POMP trial, assessing the effect of short-term oxygen supplementation, end-HOPE, with a minimum perfusion time of 120 min, on 1-year graft survival after transplantation from DBD-ECDs when compared to classic SCS. A total of 262 kidney grafts were randomized to end-HOPE (*n* = 127) or SCS (*n* = 135), finding no statistically significant differences in 1-year graft survival (92.1% vs. 93.3%; *p* = 0.71) [58]. Regarding secondary outcomes, the incidence of DGF was lower in end-HOPE when compared to SCS (23.6% vs. 28.1%), coinciding with previously published results [57], but did not reach statistical significance (*p* = 0.40) [58]. Pravisani et al. published a retrospective study of a cohort of 103 KT recipients from January 2016 to December 2020, who received a single kidney graft preserved by either end-HOPE or standard HMP with LifePort^®^ Kidney Transporter (Organ Recovery Systems, Itasca, IL, USA). They found no significant differences in terms of DGF (21.5% vs. 25.0%; *p* = 0.58), acute rejection episodes (11.7% vs. 7.7%; *p* = 0.52), or creatinine levels 1 year after transplantation (1.27 mg/dL vs. 1.40 mg/dL; *p* = 0.31) [59]. These results are in concordance with other studies [56,57,58], asserting the small effect of end-ischemic oxygen supplementation in terms of functional kidney graft results, mainly DGF.

Oxygen delivery to the perfusion circuit can be achieved through two main methods: bubble and surface oxygenation directly into the perfusion solution, maintaining a low but stable pO_2_, or with the addition of an external membrane oxygenator, allowing for higher pO_2_ levels. As mentioned previously, Darius et al. examined the effect of bubble and intermittent surface oxygenation in a porcine autotransplantation model [51]. The same group recently published their initial experience of the first-in-human use of bubble and intermittent surface oxygenation, finding no incidence of delayed graft function or acute graft rejection during follow-up, demonstrating the safety of this innovative oxygenation technique for human KT [60]. This was possible within the context of a clinical trial (NCT05430620) whose results are yet to be published.

## 4. Discussion

The COMPARE trial [54] is the only RCT comparing continuous HOPE against standard HMP. It demonstrates the significant benefits of continuous HOPE over standard HMP, with very promising functional kidney graft results, suggesting the cost-effectiveness of continuous HOPE as a means of improving graft function after KT. Compared to other published data, these results seem conflicting. The POMP trial [58] found no significant differences in 1-year graft survival, unlike the COMPARE trial [54]; however, not only is the group control for each trial different, the oxygenation strategy is also different. Jochmans et al. [54] assessed continuous HOPE while Husen et al. [58] assessed end-HOPE. Both of these differences could explain the heterogenicity of results. Nonetheless, although a small case series, Darius et al. [60] published their initial experience with intermittent oxygenation, with promising results. Until definitive results are published (NCT05430620), the difference of continuous oxygenation, intermittent oxygenation or end-ischemic oxygenation could determine the perplexity of these results. Similarly, Houtzager et al. [55] published a very limited case series (*n* = 5), with only a single kidney graft coming from a DCD. Although their results concur with safety of HOPE in humans and are in line with other studies, these results cannot suffice at reach definite conclusions.

The results from Meister et al. [56] and Pravisani et al. [59] also contradict the results from the COMPARE trial [54]. While Pravisani et al. [59] produced a study with a large number of participants (*n* = 103) and found no differences in acute graft rejection rates, DGF, or creatinine levels 1 year after transplantation, the study design was retrospective; thus, these results must be considered with caution. In the case of Meister et al. [56], they also found no differences in DGF at 6-months after transplantation, but did find an inverse association between renal resistance during and creatinine levels, coinciding with results from animal preclinical studies [49]. The Italian clinical trial by Ravaioli et al. [57] found interesting and promising results, similar to that of the COMPARE trial [54], with a 50% reduction in DGF when comparing SCS with HOPE and a 0% incidence of DGF in grafts undergoing HOPE for at least 2 h. However, the strategy for this study was end-ischemic HOPE, like the aforementioned similar studies, and its non-randomized design limits its comparison to the results of RCTs. Further RCTs focusing on continuous HOPE are needed to confirm these promising results and to fully understand how the continuous oxygenation of kidney grafts affects function and overall survival.

Although the focus of many studies are functional graft results, some studies comment on acute graft rejection rates with oxygen supplementation [54,59,60]. During ischemia, mitochondrial dysfunction generates succinate accumulation, a key component in the production of ROS during reperfusion [61,62,63]. Oxygen supplementation mitigates succinate accumulation, allowing for an increase in ATP re-synthesis during HOPE [51,64], improving graft function and long-term results. This restructuring of the IRI metabolic pathway by the addition of oxygen during preservation might explain the differences in acute graft rejection in oxygen-preserved kidney grafts. However, a long-term follow-up is needed to assess whether oxygen supplementation seems to alter acute rejection pathways, or if it acts as a protective factor against long-term graft survival.

## 5. Conclusions

Kidney graft preservation has relied on improving graft quality and optimizing functional results due to the lack of DKDs and the increasing number of waitlisted patients for KT. When understanding the pathophysiology of IRI, oxygen supplementation seems to be a possible solution to mitigate the effects of ischemic damage produced after organ procurement and the chain-like reaction after oxygenated-blood flow is restored. Oxygen supplementation has evolved from the addition of O_2_ molecules and classical SCS to the incorporation of oxygen and perfusion systems. While evidence is growing, the adoption of HOPE in clinical practice represents a promising advancement in KT preservation technology, with continuous HOPE showing the most potential for improving transplant outcomes. Further high-quality evidence from RCTs is warranted. While promising, the results of the COMPARE trial have not been replicated in other clinical trials. Only then can HOPE become an alternative preservation technique in optimizing graft function and survival, particularly for high-risk kidney donors. Nevertheless, other preservation strategies like oxygen carriers have shown potential value in optimizing kidney grafts while undergoing SCS. Though less studied than HOPE, oxygen carriers seem like an alternative preservation strategy, with pending results from RCTs that could very well place oxygen carriers as an alternative to HOPE in kidney graft preservation programs.

## Figures and Tables

**Table 1:** Human clinical studies assessing the effect of hypothermic oxygenated machine perfusion in kidney graft preservation.

Author (Year)	Country	Study Design	Population	Outcomes
Jochmans (2020) [54]	Belgium Netherlands United Kingdom	Randomized controlled phase III study Kidney Assist™	DCD ≥ 50 years old; HOPE *n* = 106 HMP *n* = 106	Significant differences in eGFR 1 year after transplantation. Less severe complications and kidney graft failure 1 year after transplantation.
Houtzager (2021) [55]	Netherlands	Case series Airdive™	*n* = 5; DBD *n* = 4 DCD *n* = 1	No differences in adverse events or safety 1 month after transplantation.
Meister (2019) [56]	Germany	Retrospective cohort study Kidney Assist™	ECD; End-HOPE *n* = 15 SCS *n* = 30	No differences in DGF 6 months after transplantation. Inverse association between renal resistance and creatinine levels, especially in grafts with PNF.
Ravaioli (2020) [57]	Italy	Non-randomized controlled phase II study	ECD; End-HOPE *n* = 10 SCS *n* = 30	No differences in adverse events or safety, PNF or graft survival 3 months after transplantation. HOPE of more than 2 h prevents DGF.
Husen (2021) [58]	Belgium Germany Hungary Netherlands United Kingdom	Randomized controlled phase III study Kidney Assist™	ECD; End-HOPE *n* = 127 SCS *n* = 135	No differences in 1-year graft survival. Lower incidence of DGF with HOPE.
Pravisani (2022) [59]	Italy	Retrospective cohort study LifePort^®^ Kidney Transporter	KT recipients (*n* = 103)	No differences in incidence of DGF, acute rejection, or creatinine levels 1 year after transplantation.
Darius (2023) [60]	Belgium	Case series LifePort^®^ Kidney Transporter	*n* = 5; Continuous surface HOPE (*n* = 3) Intermittent surface HOPE (*n* = 2)	No differences in adverse events or safety. No incidences of DGF or acute rejection.
